# Juvenile Ossifying Fibroma of the Nasal Bones: A Rare Cause of Chronic Epistaxis

**DOI:** 10.7759/cureus.45237

**Published:** 2023-09-14

**Authors:** Taqwa Drdir, Theekshitha Kamalakannan, Madiha Mohamed, Ragai Gemi, Neethu Pillai, Ahmed Elbarkouky, Bhavna Gupta

**Affiliations:** 1 General Pediatrics, Al Qassimi Women’s and Children’s Hospital, Sharjah, ARE; 2 Department of Otolaryngology, Al Qassimi Hospital, Sharjah, ARE; 3 Department of Pathology, Al Qassimi Hospital, Sharjah, ARE

**Keywords:** craniofacial bone tumor, juvenile psammomatoid ossifying fibroma, juvenile trabecular ossifying fibroma, peri-orbital cellulitis, epistaxis

## Abstract

Juvenile ossifying fibroma (JOF) is a rare type of tumor originating from the bones of the face or cranium. It usually arises in the maxilla and rarely in the mandible. The complications related to the tumor are because of local expansion and resultant effect on the nearby organs. We present the case of an eight-year-old girl with a history of headache and chronic epistaxis for the past six months who presented acutely to the hospital due to swelling, redness, and pain in both eyes, with continuous epistaxis. After investigations, she was found to have a nasal tumor that was confirmed to be JOF of the nasal bone on histopathology. Surgical management was done and the tumor was resected.

## Introduction

Juvenile ossifying fibroma (JOF) is a benign, fibro-osseous tumor that can occur in the extremities, head, and neck involving the craniofacial bones of children less than 15 years of age [1]. It is a slow-growing, locally aggressive neoplasm. Patients are typically asymptomatic and do not present until the lesion has become large enough to cause facial deformity. JOF most commonly occurs in the maxilla, followed by the mandible [2]. Rarely, lesions may also occur in the sinonasal tract, orbit, skull base, and temporal bone [3]. Our patient had an uncommon location of tumor growth involving the nasal bones.

## Case presentation

**Figure 1:**
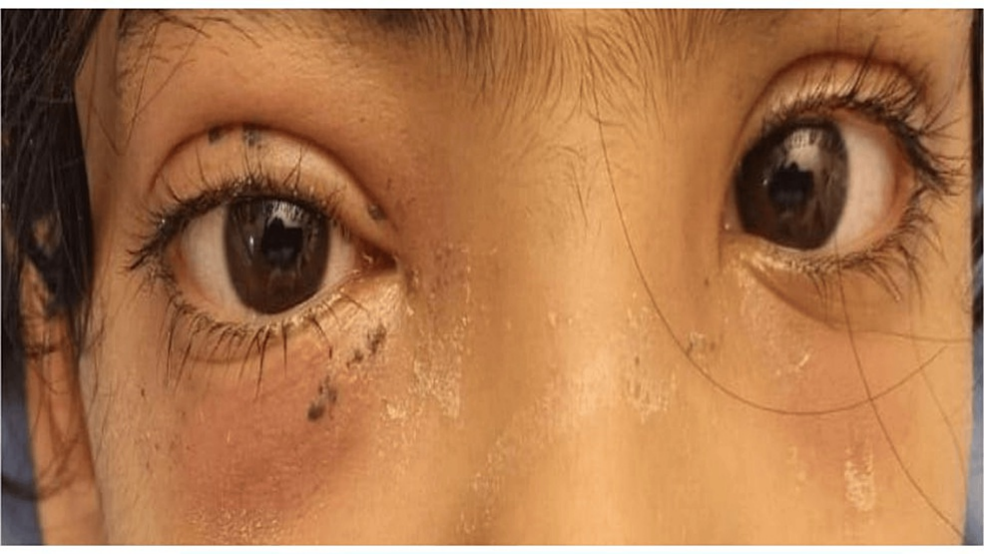
Physical findings of the eyes. Right eye mild proptosis and right-sided periorbital cellulitis were noted.

An eight-year-old, previously healthy, unvaccinated girl presented with a one-week history of fever, right-sided periorbital swelling, bilateral eye redness, and pain. She reported a history of intermittent chronic epistaxis for the preceding six months, which had increased in amount and frequency. It was associated with intermittent headache and nasal discharge for four to six months. On examination, she was afebrile, vital signs were normal, there was right eye periorbital swelling with erythema, painful right eye movement with visible blood in the nasal canal bilaterally, and a visible mass in the left nasal cavity extending to the right. She also had mild proptosis of the right eye, with no restriction of eye movements in all directions and bilateral equal-reacting pupils (Figure 1). Other systemic examinations were unremarkable. Hence, a diagnosis of a nasal mass with periorbital cellulitis was proposed.

Laboratory investigations showed leukocytosis with neutrophilia and mildly elevated C-reactive protein and erythrocyte sedimentation rate. Renal function and liver function were within normal limits. Blood culture and eye secretion cultures were negative.

**Figure 2:**
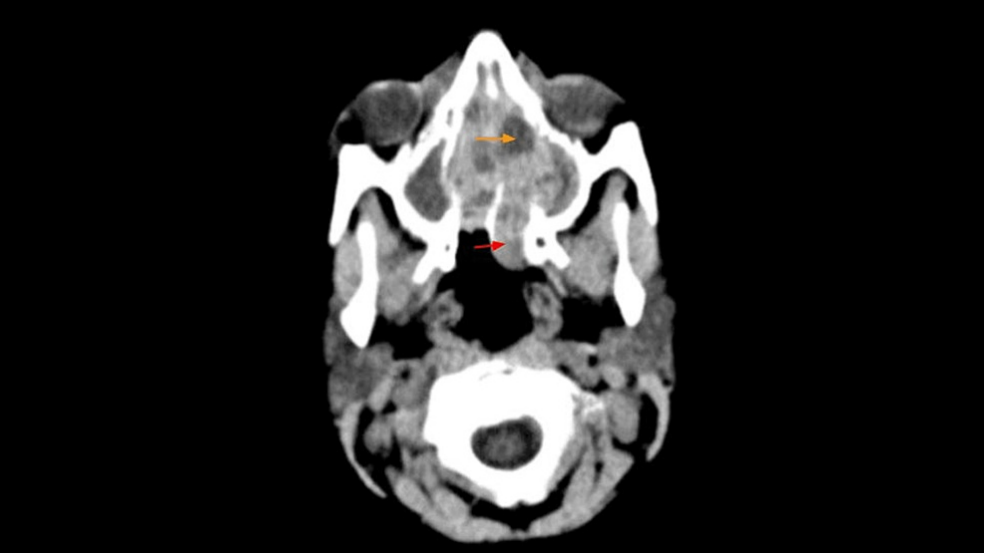
Computed tomography scan of the brain. A heterogeneous enhancement of soft tissue density seen within the nasal cavity extending through the posterior choana to the nasopharynx (red arrow) and causing some erosion in the medial wall bilaterally more in the left with extension into the left maxillary sinuses, opacified bilateral maxillary, ethmoid, and sphenoid sinuses, with right orbital media wall subperiosteal collection suggestive of abscess formation (orange arrow).

A computed tomography (CT) scan of the brain and orbit showed a tissue density (highly suggestive of vascular soft tissue) in the nasal cavity with extension into the nasopharynx and surrounding sinuses (Figure 2). A CT scan of the sinuses was also done which showed that the nasal mass was expanding the nasal cavity, with remodeling of its osseous outline, extending to the posterior choana and associated opacification of the paranasal sinuses.

**Figure 3:**
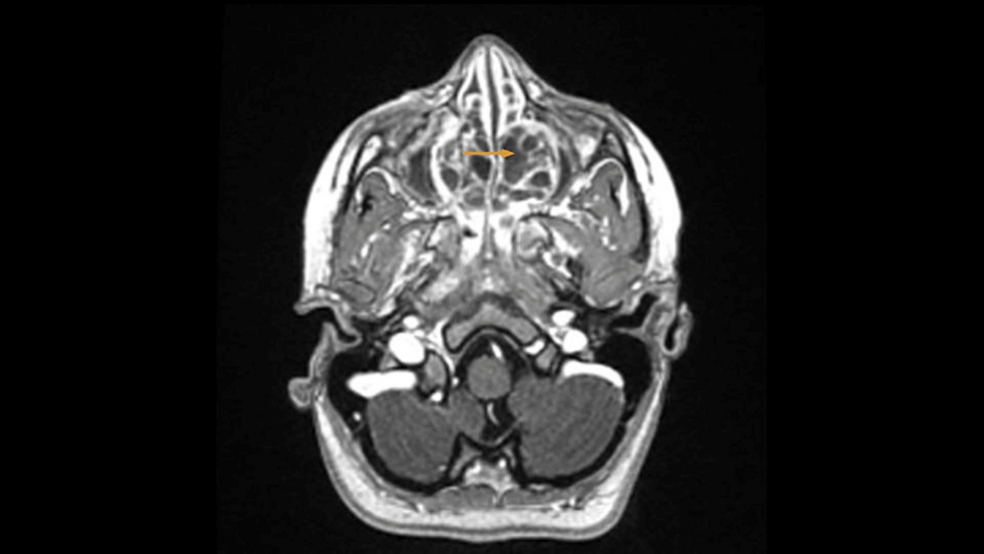
Magnetic resonance imaging of the orbit. A sizable expanding lesion within the nasal cavity extending to the left side of the nasopharynx. Associated with the bony remodeling of the maxillary sinuses’ medial walls bilaterally more evident on the left (orange arrow) suggestive of a long-standing process. The lesion shows a heterogeneous texture, with multiple highly intense foci scattered within the lesion, consistent with subacute bleeding.

Magnetic resonance imaging (MRI) of the orbit revealed the mass in the nasal cavity with a picture of chronic invasion into the surrounding structure (Figure 3).

MRI and magnetic resonance venography of the brain were unremarkable. Other imaging including chest X-ray and ultrasound of the abdomen were normal. Other specialties including Ophthalmology and Ear, Nose, and Throat were involved in the further management of the patient.

**Figure 4:**
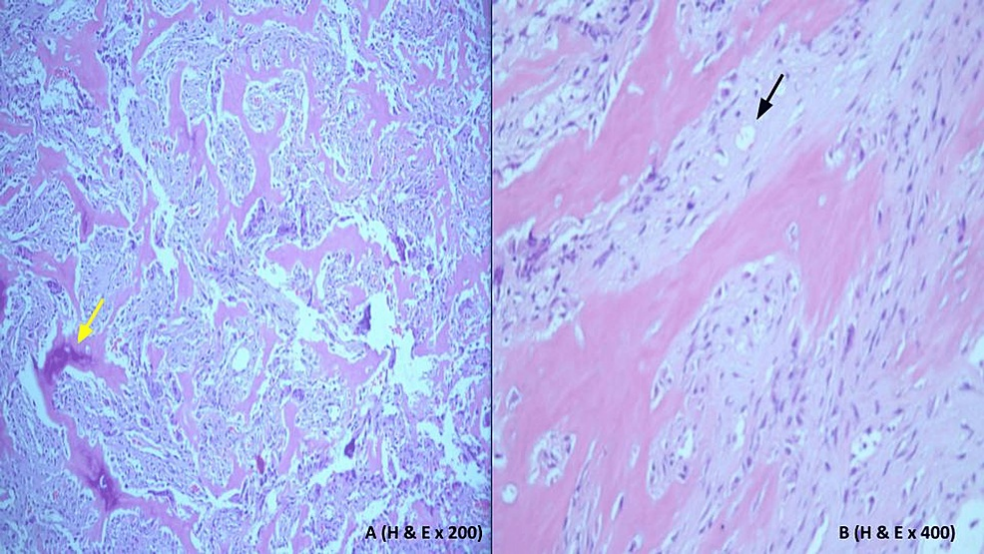
Histopathology sections. A: Cellular fibrous tissue with high vascularity and areas of hemorrhage (yellow arrow). B: Multiple sporadic areas of osteoid tissue irregular bands without a rim of osteoblasts in most of them (black arrow).

**Figure 5:**
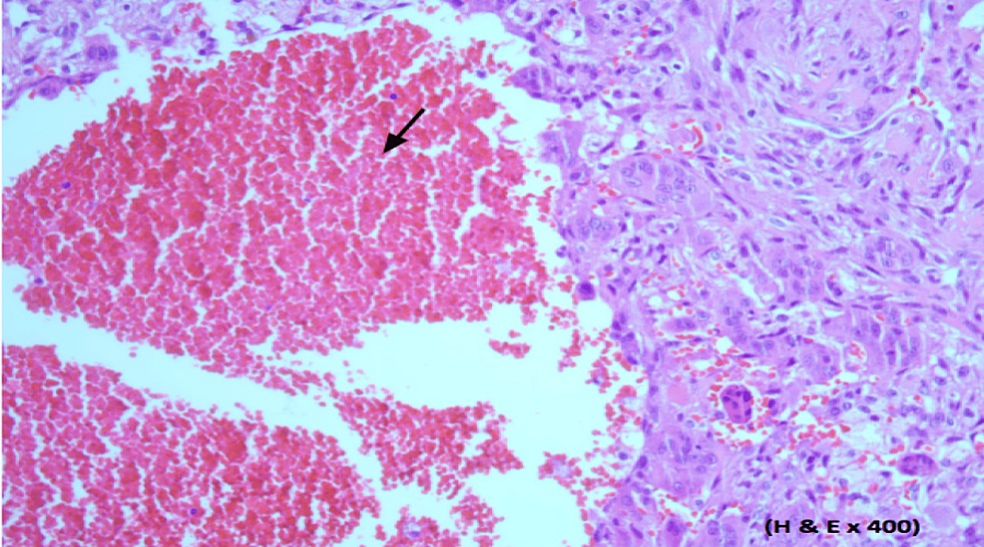
Histopathology section. Immature bony trabeculae with a rim of osteoblasts. Areas of hemorrhage (black arrow) with giant osteoclastic-like cell lining are observed giving a picture of aneurysmal bone cyst change.

She underwent an endoscopic examination of the nasal cavity bilaterally that revealed a vascular lesion, which was obstructing the left nasal cavity. A biopsy of the lesion was taken and the histopathology sections are demonstrated in Figure 4 and Figure 5.

**Figure 6:**
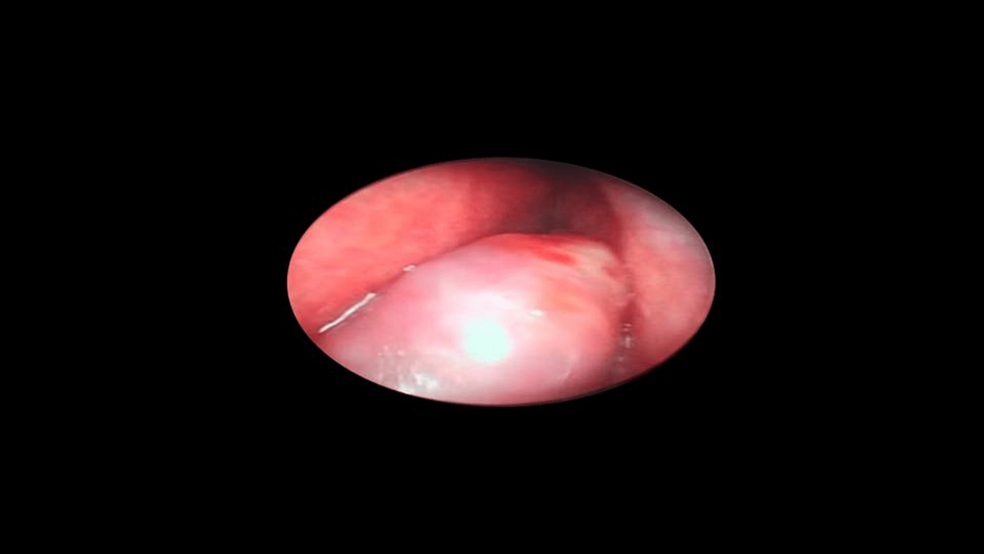
Intraoperative findings of the nasal mass. Nasal mass with a consistency of vascular soft tissue, giving no sensation of hard tissue such as bone.

The patient was operated on under general anesthesia, which showed that the nasal mass (Figure 6) involved both nasal cavities, eroding the posterior end of the septum and extending to the choanae. The mass was removed under image guidance from both the nasal cavity and choana.

She received intravenous antibiotics in the hospital for four weeks. Once epistaxis had improved and periorbital cellulitis resolved, she was discharged with close follow-up. After three months, she had a recurrence of the mass as she presented with epistaxis, bilateral eye pain, and headache. She was managed by a multidisciplinary team including otolaryngology, oncology, and general pediatrics. The mass was fully resected and she was discharged in good condition. She is doing well and remains symptom-free on follow-up two months after the second surgery.

## Discussion

JOF is a rare tumor that appears in the craniofacial bones and has been reported in children less than 15 years old. It is a benign, fibro-osseous, locally aggressive tumor that usually increases in size, expands to surrounding structures, and may lead to facial asymmetry [1]. Histopathological types include trabecular and psammomatoid. Differences have been reported in the distribution of bone involvement. The psammomatoid type usually affects the orbital bones, orbit, and paranasal sinuses, whereas the trabecular type usually affects the bones of the jaw [4]. Prognostically, psammomatoid JOF has an aggressive course with a tendency to recur compared to trabecular JOF [5].

JOF usually does not cause any symptoms on its own, rather the symptoms arise due to local expansion which is usually painless but can cause pressure and sometimes deformities to the surrounding organs. Cases of this tumor have been reported in the maxilla and rarely in the mandible. Maxillary growths usually involve the nasal cavity or the orbit. When extension occurs in the nasal cavity, symptoms can arise due to obstruction of the nearby sinuses presenting with sinusitis. In addition, epistaxis can occur due to local expansion, as was the case with our patient. If the tumor involves the orbit, the patient can present with dystopia, proptosis, and painful eyes [1]. In our case, the patient had eye pain, proptosis, and periorbital cellulitis. Similarly, a case report of a 15-year-old girl suffering from unilateral proptosis and obstruction in the same side nostril was diagnosed with JOF with orbital and intracranial extension and required multiple surgeries [2].

Management of this tumor is by surgical excision. Conservative approaches such as curettage are not optimal as this tumor has a very high chance of recurrence [4]. However, the tumor resection can be limited by its extent into the surrounding vital organs as well as the cosmetic outcomes. Reconstructive surgery is usually delayed as the patient should be assessed in the long term to exclude any recurrence. Furthermore, radiotherapy has been proven to be ineffective as the tumor is radioresistant [1]. A favorable prognosis of the tumor, unless a recurrence of the tumor occurs, has been reported in the literature [3].

## Conclusions

JOF is a rare clinical entity that is locally aggressive in nature and has an unpredictable recurrence, which poses a challenge and warrants awareness among clinicians. The majority of the cases are asymptomatic and present once the complications occur. As in our case, the patient had a common symptom of epistaxis but given its chronicity should warrant suspicion of an underlying pathology. Furthermore, this tumor is mainly found in the maxilla but in rare cases like ours can grow in other craniofacial bones such as the nasal bone. Biopsy aids in confirming the diagnosis, and surgical excision remains the mainstay of treatment.
